# Academy of Medicine Notes

**Published:** 1894-07

**Authors:** 


### Academy of Medicine Notes.

The general attendance at the Academy and section meetings, during the past year, has been very satisfactory, and the meetings, as a whole, have been very instructive and beneficial. Still, great improvements can be made, both by the officers and members, and the meetings of 1894-95 should be superior to the ones of 1893 and '94. The Academy is a success and the profession of Buffalo should give it a warm and hearty support.

At the last stated meeting of the Academy, the following amendment to the constitution and by-laws was adopted :

*Resolved*, That the council of the Academy shall act and perform the duties of the committee on legislation of the Academy of Medicine.

Owing to the absence from the city of the president, the address at the annual meeting, June 26th, was delivered by Mr. George W. Rafter, of Rochester, on Intermittent Filtration in its Application to Domestic Filters.

THE Academy elected Dr. P. W. Van Peyma president; Dr. Eugene A. Smith, treasurer; Dr. A. L. Benedict, secretary, and Dr. H. R. Hopkins, trustee, at the annual meeting, June 26, 1894.

---

THE section on surgery elected Dr. Marcell Hartwig president; Dr. H. Mynter, vice-president, and Dr. E. J. Gilray, secretary, at its last meeting.

---

THE section of anatomy, physiology and pathology elected Dr. S. Y. Howell president and Dr. F. T. Metcalfe secretary for the ensuing year.

---

DRS. HURD, Matzinger and Bussman were elected fellows of the Academy at the meeting held June 19, 1894.

---

THE sections of the Academy have adjourned until September.

